# Oxidative stress biomarkers and acetylcholinesterase activity in human erythrocytes exposed to clomazone (in vitro)

**DOI:** 10.2478/v10102-011-0023-9

**Published:** 2011-09

**Authors:** Adriana Santi, Charlene Menezes, Marta Maria F. Duarte, Jossiele Leitemperger, Thais Lópes, Vania L. Loro

**Affiliations:** Departamento de Química, Centro de Ciências Naturais e Exatas, Programa de Pós-Graduação em Bioquímica Toxicológica, Universidade Federal de Santa Maria, RS, Brazil

**Keywords:** clomazone, erythrocytes, oxidative stress, acetylcholinesterase

## Abstract

The aim of this study was to investigate the effect of clomazone herbicide on oxidative stress biomarkers and acetylcholinesterase activity in human erythrocytes in *in vitro* conditions. The activity of catalase (CAT), superoxide dismutase (SOD) and acetylcholinesterase (AChE), as well as the levels of thiobarbituric acid reactive substances (TBARS) and reduced glutathione (GSH) were measured in human erythrocytes exposed (*in vitro*) to clomazone at varying concentrations in the range of 0, 100, 250 and 500 µg/L for 1 h at 37 °C.TBARS levels were significantly higher in erythrocytes incubated with clomazone at 100, 250 and 500 µg/L. However, erythrocyte CAT and AChE activities were decreased at all concentrations tested. SOD activity was increased only at 100 µg/L of clomazone. GSH levels did not change with clomazone exposure. These results clearly showed clomazone to induce oxidative stress and AChE inhibition in human erythrocytes (*in vitro*). We, thus, suggest a possible role of ROS on toxicity mechanism induced by clomazone in humans.

## Introduction

The widespread use of pesticides in agriculture results in continuous exposure of human populations. Low-level exposure to pesticides is known to produce a variety of biochemical changes such as target cell/receptor binding, protein and DNA adduct formation, as well as induction or inhibition of enzymes (López *et al.*, 2007). Clomazone (2-[(2-chlorophenyl) methyl]-4, 4-dimethyl-3-isoxazolidinone), which is a soil-applied herbicide, has been reported to interfere with chloroplast development and to reduce or prevent accumulation of plastid pigments in susceptible species (Ferhatoglu & Barrett, 2006). It belongs to the class of isoxazolidinones and is widely used against weeds in paddy rice fields in Southern Brazil (Crestani *et al.*, 2007).

Biochemical and physiological functions of red blood cells (RBC) can be affected by pesticides. Erythrocytes are particularly sensitive to oxidative damage due to the presence of high polyunsaturated fatty acid content in their membranes and high cellular concentrations of oxygen and haemoglobin (Hgb) (Prasanthi *et al.*, 2005; Mansour & Mossa, 2009). In addition, it is speculated that oxidative stress in erythrocytes may lead to significant alterations in their structural conformation, which may compromise effective blood flow, oxygen uptake and release (Prasanthi *et al.*, 2005). Lipid peroxidation (LPO), which is the major contributor to the loss of cell function, as well as DNA damage, enzyme inactivation, and hormone oxidation are indicators of oxidative cell damage (Ruas *et al.*, 2008). LPO, in particular, has been suggested to be one of the mechanisms of pesticide-induced toxicity (Mansour & Mossa, 2009).

Erythrocytes are well equipped with several biological mechanisms which defend them against intracellular oxidative stress including antioxidant enzymes, such as superoxide dismutase (SOD) and catalase (CAT) (Prasanthi *et al.*, 2005). SOD enzymes belongs to metalloenzymes, which transform superoxide anions (O_2_−) into less reactive species, namely molecular oxygen (O_2_) and hydrogen peroxide (H_2_O_2_). The H_2_O_2_ formed by SOD activity is decomposed to H_2_O and O_2_ by CAT and/or glutathione peroxidase (GPx) in the presence of reduced glutathione (GSH) (Ruas *et al.*, 2008). CAT is ubiquitously present in a wide range of aerobic cell types and in red blood cells where it is found as a soluble protein that may protect against peroxidation of haemoglobin. Because of the important role of CAT in the scavenging process of H_2_O_2_, the investigation of possible changes in the activity of this enzyme under the influence of commonly used herbicides seems to be essential (Bukowska *et al.*, 2000).

Acetylcholinesterase (AChE) in erythrocytes is one of the typical extraneural AChE enzymes. AChE has an essential role in acetylcholine-mediated neurotransmission. It is present in the cholinergic synapses in the central nervous system as well as in neuromuscular synapses where it rapidly hydrolyzes acetylcholine (Igisu *et al.*, 1994; Jha & Rizvi, 2009). The determination of AChE activity in blood is a great consideration in the diagnosis of poisonings caused by reversible and irreversible inhibitors of this enzyme including pesticides (Bukowska & Hutnika, 2006).

GSH is the most abundant non-protein thiol in cells participating in several processes, including synthesis of DNA and proteins, regulation of enzyme activity and inter- and intracellular transport. In human erythrocytes, about 99% of the existing glutathione is in the reduced form under normal physiological conditions, where it affects the scavenging of all functions, including free radical reactions (Bukowska, 2003; Celik & Suzek, 2009). The consequence of oxidative stress in cells may be a decrease of GSH and increase of its oxidized form (GSSG) (Luperchio *et al.*, 1996)

Considering the fact that erythrocytes are susceptible to oxidative stress induced by pesticide as well as that studies describing the role of reactive oxygen species (ROS) in clomazone toxicity are limited, the aim of this study was to investigate the effect of clomazone on oxidative stress biomarkers and AChE activity in human erythrocytes (*in vitro).*

## Material and methods

### Chemicals

The herbicide clomazone {2-[(2-cholorophenyl)-methyl]-4,4-dimethyl-3-isoxazolidinone} used in this study was obtained commercially from the FMC Corporation (Gamit; 50% purity, Philadelphia, EUA). Malondialdehyde (MDA), 2-thiobarbituric acid (TBA), bovine serum albumin, hydrogen peroxide (H_2_O_2_) and other reagents were obtained from Sigma Chemical Co. (St. Louis, MO, USA).

### Preparation of erythrocytes

Blood samples were obtained from twelve healthy donors (50% female and 50% male; age, mean ± SD (years): 40.7 ± 9.84) from Labimed (Santa Maria, RS, Brazil). The volunteers were non-smokers, non-alcohol drinkers, and under no medication or food supplement intake. Blood samples were collected after a 12-h overnight fasting by venous puncture into gray top Vacutainers® (BD Diagnostics, Plymouth, UK) tubes with sodium fluoride plus potassium oxalate. Specimens were routinely centrifuged at 3 000 rpm for 10 min at 4 °C, and the plasma and buffy coat were removed. The erythrocytes were washed three times with cold isotonic saline and centrifuged. After the final wash, the packed erythrocytes were resuspended in phosphate buffer (0.1 mol/L, pH 7.4) at 1:9 dilution and used for incubations. The protocol was approved by the Human Ethics Committee of the Federal University of Santa Maria (number 0291.0.243.000-09).

### Treatment of erythrocytes

Erythrocytes suspended in phosphate buffer were exposed to clomazone at varying concentrations in the range of 0, 100, 250 and 500 µg/L for 1h at 37 °C (with continuous mixing). There are not sufficient data available on the cytotoxic effects of clomazone using erythrocyte as a model. The concentration usually recommended in rice fields is from 0.4 to 0.7 mg/L (Rodrigues & Almeida, 1998) and thus we chose clomazone concentrations of 0–500 µg/L for the experiments. Samples of erythocytes and phosphate buffer without herbicide were used as controls. Both the samples exposed to clomazone and controls were stored at −20 °C until biochemical analysis. All the experiments were repeated ten times under the same conditions.

### Determination of lipid peroxidation

Lipid peroxidation was assessed by measuring TBARS in erythrocytes, according to a modified method of Jentzsch *et al.* (1996). Briefly, 0.2 mL of erythrocytes was added to the reaction mixture containing 1 mL of 1% ortho-phosphoric acid, 0.25 mL alkaline solution of thiobarbituric acid-TBA (final volume 2.0 mL) followed by 45 min heating at 95 °C. After cooling, samples and standards of malondialdehyde were read at 532 nm against the blank of the standard curve. The results were expressed as nmol MDA/mL erythrocytes.

### Catalase activity

CAT activity in erythrocyte lysate was determinated according to the method of Aebi (1984). The method is based on the decomposition of H_2_O_2_ by catalase. An aliquot (0.02 mL) of erythrocyte lisate was homogenised in potassium phosphate buffer, pH 7.0. The spectrophotometric determination was initiated by the addition of 0.1 mL in an aqueous solution of hydrogen peroxide 0.3 mol/L. The reduction rate of H_2_O_2_ was followed at 240 nm for 1 min. Catalase activity was expressed in µmol/mg protein /minute.

### Superoxide dismutase activity

SOD activity measurement is based on the inhibition of the radical superoxide reaction with adrenaline as described by McCord & Fridovich (1969). In this method, SOD present in the sample competes with the detection system for superoxide anion. A unit of SOD is defined as the amount of enzyme that inhibits the rate of adrenaline oxidation by 50%. Adrenaline oxidation leads to the formation of the coloured product, adrenochrome, which is detected by spectrophotometer. SOD activity is determined by measuring the rate of adrenochrome formation, observed at 480 nm, in a reaction medium containing glycine-NaOH (50 mmol/L, pH 10) and adrenaline (1 mmol/L). The results were expressed as U/mg protein.

### Acetylcholinesterase activity

AChE activity was assayed by the method of Ellman *et al.* (1961). According to this method, acetylthiocholine (AcSCH) is hydrolyzed by AChE to acetic acid and thiocholine. The catalytic activity is measured by the increase of the yellow anion, 5-thio-2-nitrobenzoate, produced from thiocholine when it reacts with 5,50-dithio-bis-2-nitrobenzoic acid (DTNB). AChE activity was expressed in µmol of AcSCH hydrolyzed/ min/ mg protein.

### Reduced glutathione

Reduced glutathione was assayed by the method of Ellman (1959). Aliquots (0.1 mL) of erythrocytes were added to a phosphate buffer 0.3 mol/L (0.85 mL), pH 7.4 and the reaction was followed at 412 nm after the addition of 0.05 mL of 10 mmol/L 5-5’-dithio-bis(2-nitrobenzoic acid) (DTNB). Results were expressed as µmol/ml of erythrocytes.

### Protein determination

Protein was determined by the Comassie blue method using bovine serum albumin as standard. Absorbance of samples was measured at 595 nm (Bradford, 1976).

### Statistical analysis

All data were expressed as mean ± standard deviation (SD). Data were analysed statistically using analysis of variance followed by the Duncan multiple test. Statistical significance was assumed at *p*<0.05. Data analysis was performed with Statistica 6.0 (StatSoft, Inc, Tulsa, OK, USA, 2001).

## Results

Table 1 shows oxidative stress biomarkers and acetylcholinesterase activity in erythrocytes incubated with clomazone during 1h. TBARS levels were significantly increased in all clomazone concentrations used compared to control value. On the contrary, a significant reduction in the activity of AChE was observed at all clomazone concentrations. No statistically significant changes were observed in GSH levels as compared to control group. The lowest dose of clomazone (100 µg/L) appeared to result in statistically significant induction of SOD activity when compared to control. The activity of erythrocyte CAT decreased when red blood cells were exposed to clomazone (Table 2).

**Table 1:** Oxidative stress biomarkers and acetylcholinesterase activity in erythrocytes incubated with clomazone during 1 h.

Biomarker	Control	100 µg/L	250 µg/L	500 µg/L
TBARS (nmol MDA/mL)	17.02±3.61^a^	26.96±8.39^b^	34.07±7.09	31.21±7.60^b,c^
AchE (µmol AcSCH min/mg protein)	0.86±0.13^c^	0.61±0.03^b^	0.48±0.10^a^	0.39±0.04^a^
GSH (µmol/ml)	1.31±0.12^a^	1.44±0.37^a,b^	1.27±0.10^a,c^	1.36±0.04^a^

Data are expressed as mean±SD of ten experiments. Duncan's multiple range test: groups that show different letters are statistically different (*p*≤0.05).

**Table 2:** Erythrocyte superoxide dismutase (SOD) and catalase (CAT) activities in human erythrocytes incubated with clomazone for 1 h.

	Activity of SOD (U/mg protein)	Activity of CAT (µmol/mg protein/min)
Control	3.58±0.96^a^	34.28±5.18^a^
100 µg/L	6.31±0.50^b^	30.28±5.32^b^
250 µg/L	3.14±1.16^a^	29.77±5.76^b^
500 µg/L	2.85±0.74^a^	25.83±4.21^c^

Data are expressed as mean±SD of ten experiments. Duncan's multiple range test: groups that show different letters are statistically different (*p*≤0.05).

## Discussion

Erythrocytes are a convenient model to understand the membrane oxidative damage induced by various xenobiotic pro-oxidants (Mansour *et al.*, 2009).The treatment with clomazone (*in vitro*) showed increased LPO in human erythrocytes. Polyunsaturated fatty acids of the membrane, an oxygen-rich environment, as well as iron-rich haemoglobin make red cells susceptible to peroxidative damage. ROS initiates LPO reactions that lead to loss of membrane integrity and, consequently, death of the cell. Malondialdehyde (MDA), a highly reactive bifunctional molecule, is an endproduct of membrane LPO. MDA has been shown to cross-link erythrocyte phospholipids and proteins. This process results in impairment of the membrane-related functions, leading ultimately to diminished survival (Çimen, 2008). The increased TBARS levels found in this study may have resulted from an increase of free radicals as a result of stress condition generated by erythrocyte herbicide exposure. These findings are consistent with results of several other recent investigations (Prasanthi *et al.*, 2005; Duchnowicz *et al.*, 2005).

Enzymes for preventing oxidative denaturation in erythrocytes include SOD and CAT. Superoxide anion is converted into O_2_ and H_2_O_2_ by SOD, a ubiquitous metal-containing enzyme (Çimen, 2008). Physiologically, erythrocytes are well protected against ROS by abundant Cu, Zn-SOD, which scavenge free radical thus preventing methaemoglobin (metHgb) formation (Dumaswala *et al.*, 1999). In this study we observed higher SOD activity at 100 µg/L of clomazone. The increased SOD activity may be a consequence of cellular-oxidative damage due to pesticide exposure in this concentration. Moreover, in higher doses of clomazone, an inhibition tendency of SOD activity was observed, possibly due to toxic effects of higher doses of the herbicide. Therefore, oxidative stress is likely to be potentially related to SOD activity damage in *in vitro* conditions. Damage in SOD can significantly affect the defensive mechanisms against free radical attack in the living cell, such as human erythrocytes (Bukowska, 2003). On the other hand, the activity of erythrocyte SOD decreased with the increasing dose of 2, 4-D and 2, 4-DCP, for both herbicides (Bukowska, 2004).

CAT activity significantly decreased in the erythrocytes after clomazone exposure in a dose-dependent manner. Low CAT activity could also be attributed to enzyme inactivation by ROS-induced damage to proteins (Nelson *et al.*, 2006). CAT, a soluble protein in erythrocytes, plays a role in the decomposition of hydrogen peroxide to give H_2_O. On balance, our results are in accordance with other recent investigations that showed phosalone, chlorpyrifos-ethyl and phenoxyherbicide metabolites to cause a decrease in CAT activity (Gultekin *et al.*, 2001; Altuntas *et al.*, 2002; Bukowska *et al.*, 2000).

In the present study, the erythrocyte AChE activity was markedly inhibited after clomazone treatment. AChE in blood cells is biochemically identical to the enzyme contained in neurons and reveals lower individual dispersion as well as higher resistance towards external factors. Erythrocyte AChE plays an important role in the preservation of the integrity of the red cell. Markedly reduced erythrocyte AChE activity was demonstrated in cases of paroxysmal nocturnal haemoglobinuria (PNH) (Auditore & Hartmann, 2004).Previous studies showed a correlation between AChE inhibition in blood and inhibition in target tissues (Kale *et al.*, 1999, Igisu *et al.*, 1994). Clomazone significantly inhibited AChE activity in brain and muscle of silver catfish (Rhamdia quelen) exposed to 5, 10 and 20 mg/L for 96 h (dos Santos Miron *et al.*, 2005).The same result was found by Bukowska *et al.* (2007), where AChE inhibition was observed in human erythrocytes incubated with 3-(dimethylamino) phenol, a derivative of phenoxyherbicides.

In summary, the present study demonstrated an increase of oxidative stress in human erythrocytes exposed to clomazone herbicide (*in vitro*). This study clearly showed an inhibition of AChE activity, presenting this enzyme as a good indicator of intoxication of erythrocytes by pesticides. Nevertheless, in addition to *in vitro* studies, larger clinical studies are required to better understand clomazone toxic mechanisms in humans.
